# Neonicotinoid Residues in Tea Products from China: Contamination Patterns and Implications for Human Exposure

**DOI:** 10.3390/toxics13070550

**Published:** 2025-06-29

**Authors:** Yulong Fan, Hongwei Jin, Jinru Chen, Kai Lin, Lihua Zhu, Yijia Guo, Jiajia Ji, Xiaming Chen

**Affiliations:** 1 Public Health Service Center, Bao’an District, Shenzhen 518105, China; fanyul117@163.com (Y.F.); sgzjk209@126.com (J.C.); zhuivy810@126.com (L.Z.); yijiaguo@foxmail.com (Y.G.); 2 School of Public Health, Sun Yat-Sen University, Guangzhou 510080, China; 3 Shenzhen Guangming District Center for Disease Control and Prevention, Shenzhen 518107, China; jhw790216@163.com; 4 Shenzhen Center for Disease Control and Prevention, Shenzhen 518055, China; diaozhu3922855@126.com (K.L.); jiajia0929@sohu.com (J.J.)

**Keywords:** neonicotinoids, tea, residue characteristics, tea processing, human exposure, health risk

## Abstract

Neonicotinoids (NEOs) are a class of systemic insecticides widely used in agriculture owing to their high efficacy and selectivity. As one of the most globally consumed beverages, tea may represent a potential dietary source of pesticide residues. However, limited research has examined NEO contamination in tea and its implications for human exposure, highlighting the need for further investigation. Therefore, this study comprehensively evaluated the residue characteristics, processing effects, and human exposure risks of six NEOs—dinotefuran (DIN), imidacloprid (IMI), acetamiprid (ACE), thiamethoxam (THM), clothianidin (CLO), and thiacloprid (THI)—in Chinese tea products. According to the findings, the primary pollutants, ACE, DIN, and IMI, accounted for 95.65% of the total NEO residues in 137 tea samples, including green, oolong, white, black, dark, and herbal teas. The highest total target NEO (∑_6_NEOs) residue level was detected in oolong tea (mean: 57.86 ng/g). Meanwhile, IMI exhibited the highest residue level (78.88 ng/g) in herbal tea due to the absence of high-temperature fixation procedures. Concentrations of DIN in 61 samples (44.5%) exceeded the European Union’s maximum residue limit of 10 ng/g. Health risk assessment indicated that both the chronic hazard quotient (cHQ) and acute hazard quotient (aHQ) for adults and children were below the safety threshold (<1). However, children required special attention, as their exposure risk was 1.28 times higher than that of adults. The distribution of NEO residues was significantly influenced by tea processing techniques, such as full fermentation in black tea. Optimizing processing methods (e.g., using infrared enzyme deactivation) and implementing targeted pesticide application strategies may help mitigate risk. These results provide a scientific foundation for enhancing tea safety regulations and protecting consumer health.

## 1. Introduction

Neonicotinoids (NEOs) represent the most rapidly expanding and widely utilized class of insecticides in recent decades due to their potent insecticidal activity, broad-spectrum efficacy, and relatively low mammalian toxicity [1,2]. NEOs are employed in agricultural practices to manage crop pests through methods such as foliar application, seed treatment, and soil incorporation [3]. To disrupt acetylcholine-mediated neurotransmission and ultimately cause excitation, paralysis, or death, NEOs function as insecticides by activating nicotinic acetylcholine receptors (nAChRs) in the insect central nervous system [4]. Since their development in the 1980s [5], NEOs have experienced substantial global growth in usage. Currently accounting for over a quarter of the insecticide market share and more than 30% of the global pesticide market [6], NEOs generate annual sales exceeding USD 3.5 billion [7], making them the most widely used class of insecticides worldwide. Despite their high target selectivity, NEOs still may pose risks to non-target organisms due to environmental residues resulting from chronic or high-dose exposure through passive ingestion or dermal absorption [2]. These substances have been detected in various environmental media and non-target organisms, including earthworms, nematodes [8,9], and bees [1,10]. Additionally, human health-related studies have indicated that NEO residues have been detected in crops, fruits, vegetables [11,12], and even human breast milk [13].

Major NEOs include dinotefuran (DIN), imidacloprid (IMI), acetamiprid (ACE), thiamethoxam (THM), clothianidin (CLO), and thiacloprid (THI). Animal and in vitro studies have demonstrated that exposure to NEOs at doses exceeding environmental concentrations can induce various toxic effects in mammals [14]. The distinctive characteristic of NEOs’ toxicity is their ability to bind to the α4β2 subtype of nAChRs, which is predominant in mammals. Changes in the density of this receptor subtype have been associated with several central nervous system disorders, including Alzheimer’s disease, Parkinson’s disease, schizophrenia, and depression [15]. Additionally, NEO insecticides may adversely affect mammalian reproductive organs, leading to testicular underdevelopment, impaired spermatogenesis, reduced sperm quality, and morphological alterations in the ovaries [16]. For both urban and rural populations, dietary intake is likely the primary pathway through which environmental NEOs enter the human body [17]. In recent years, nations and regions have implemented laws and restrictions to regulate the use or registration of NEOs due to their adverse effects on non-target organisms. The European Union prohibited the outdoor use of several NEO-based insecticides in 2018 [18], and the U.S. Environmental Protection Agency (EPA) revoked the registration of 12 NEO-containing products in 2019 [19]. To detect NEOs in food products, China has also established technical requirements and maximum residue limits. For example, the permitted levels in tea were set at 5 mg/kg for DIN and 0.5 mg/kg for IMI in 2021 [20]. As one of the most populous and agriculturally productive nations in the world, China both produces and consumes substantial amounts of NEO compounds. This widespread use results in non-negligible health risks to the Chinese population [21], particularly through dietary exposure. Therefore, a comprehensive understanding of NEO residues in food is essential for assessing and mitigating potential human health risks.

Tea, the world’s second most consumed non-alcoholic beverage, accounts for approximately 25% of global agricultural commodity trade [22]. Its chemical constituents demonstrate diverse bioactivities, including antioxidant, anticancer, hepatoprotective, and antidiabetic properties [22], making tea a significant component of the Chinese diet. Based on the degree of fermentation, tea can be classified into six types: green tea (non-fermented), white tea (semi-fermented), yellow tea (semi-fermented), oolong tea (semi-fermented), black tea (fully fermented), and dark tea (post-fermented) [23]. Herbal tea, commonly referred to as tisane, is made from edible parts of plants—such as leaves, flowers, fruits, or roots—and consumed in a manner like traditional tea, typically through steeping or boiling [24]. Common examples include chrysanthemum tea and jasmine tea [25,26]. The tea market has expanded in recent years, due to the increasing popularity of herbal teas. Demand for these products has risen significantly owing to their purported anti-inflammatory, antioxidant, antithrombotic, antihypertensive, and hypoglycemic properties [27]. In China, NEOs are routinely utilized to mitigate pest damage in tea cultivation [28]. As of February 2020, ten NEO compounds were officially approved and registered for agricultural use across the nation [29]. The potential health risks posed by NEO residues in tea products have garnered increasing public concern. Due to their strong polarity, NEO compounds readily leach from dried tea leaves into the brewed tea, making ingestion a primary route of human exposure to these residues [30]. Differences in tea processing methods may result in variations in NEO residue levels. For example, the high temperatures during the withering stage (40–50 °C) may affect the degradation rate of NEOs, while baking at 100–120 °C may further modify their chemical structures [31]. The transfer characteristics of NEOs from tea leaves to infusion have been systematically investigated in previous studies [28,32]. However, research focusing on the impact of processing methods on the distribution of NEO residues remains limited. Most investigations have concentrated on the six major tea types, whereas studies on NEOs and related residues in reprocessed or herbal teas are scarce, with limited characterization of contamination profiles in these products.

To address the current data gap, this study collected 137 representative tea product samples from four major tea-producing regions in China. The samples included non-fermented green tea, semi-fermented white tea and oolong tea, fully fermented black tea, post-fermented dark tea, and herbal (substitute) teas. Six NEOs—DIN, IMI, ACE, THM, CLO, and THI—were quantified in tea samples using high-performance liquid chromatography coupled with tandem mass spectrometry (HPLC-MS/MS). The objectives of this study were to: (1) assess NEO residue levels in six types of Chinese tea; (2) investigate how different tea processing methods influence NEO residue distribution; and (3) evaluate potential health risks associated with NEO intake through tea consumption while providing consumption guidance for consumers.

## 2. Materials and Methods

### 2.1. Chemicals and Reagents

Reference standards of DIN, THM, CLO, IMI, ACE, and THI, along with their corresponding internal standards, were obtained from Sigma-Aldrich (St. Louis, MO, USA). Acetonitrile and methanol (HPLC grade) were purchased from Merck (Darmstadt, Germany). Formic acid (>98% purity), acetic acid (>95% purity), magnesium sulfate (MgSO_4_), and sodium citrate were sourced from CNW (ANPEL, Shanghai, China). Agilent Technologies (Santa Clara, CA, USA) provided the dispersive solid-phase extraction sorbents (50 mg PSA, 50 mg C18, 150 mg MgSO_4_, and 10 mg GCB). A Millipore purification system was utilized to purify the water used in each experiment (Millipore, Billerica, MA, USA).

### 2.2. Sampling and Sample Preparation

To minimize potential sampling bias, a total of 137 tea samples representing different tea types were collected, including green tea (*n* = 20), white tea (*n* = 15), oolong tea (*n* = 59), black tea (*n* = 21), dark tea (*n* = 15), and herbal tea (*n* = 7). The samples utilized in this study were obtained from the four principal tea-producing regions in China: Fujian, Yunnan, Guangdong, and Henan [28]. All samples were purchased from physical retail stores. During the sample selection process, a comprehensive range of market segments—including high-, medium-, and low-end products—was incorporated. The criteria for tea quality selection were as follows: The tea exhibited no visible signs of deterioration, such as mold, off-odors, or abnormal discoloration. Production and expiration dates confirmed that all samples were within their shelf life. The packaging remained intact, with no damage or leakage, ensuring that the samples were not compromised during storage or transportation. To ensure sample representativeness and minimize potential bias, we employed a rigorous randomization strategy: tea samples were randomly selected from markets within designated regions, with individual packages randomly purchased from retail outlets and production batches randomly selected from manufacturers. Three markets were selected per province, with no fewer than 10 samples collected from each market. Additional details regarding the tea samples are provided in Table S1. After grinding and sieving, the samples were stored in airtight bags at 4 °C until further analysis.

Before analysis, 500 mg of each tea sample was weighed into a plastic tube and mixed with 300 mg of MgSO_4_, 50 mg of sodium citrate, and an isotope-labeled internal standard. Subsequently, 1 mL of acetonitrile was added, and the mixture was soaked for 1 h. Acetonitrile containing 0.1% acetic acid was then added to adjust the final volume to 2 mL. After 10 min of ultrasonic extraction, the sample was centrifuged for 20 min at 5000 rpm (acetonitrile-phase supernatant). During purification, 1 mL of the supernatant was transferred to a tube containing dispersive solid-phase extraction sorbents, followed by shaking for 5 min and centrifugation at 8000 rpm for 20 min. The resulting supernatant was collected and evaporated to near dryness under a stream of high-purity nitrogen. The residue was reconstituted in 1 mL of 30% acetonitrile in distilled water, filtered through a 0.2 μm membrane, and stored at −20 °C until analysis.

### 2.3. Instrumental Analysis

NEOs in tea samples were analyzed using a Benly SiO6512 QuEChERS automated sample preparation system (Beijing Ability Technology Co., Ltd., Beijing, China) coupled with a Waters TQ-XS ultra-performance liquid chromatography–tandem mass spectrometry (UPLC-MS/MS) system (Thermo Scientific, Waltham, MA, USA). The mobile phase comprised an aqueous solution of 2 mM NH_4_FA with 0.01% FA (solvent A) and methanol containing 2 mM NH_4_FA and 0.01% FA (solvent B). The injection volume was 0.5 μL. The gradient elution program was as follows: 0–1.0 min, 3% B; 1.0–5.0 min, 3–50% B; 5.0–7.5 min, 50–70% B; 7.5–7.6 min, 70–3% B; and 7.6–10.0 min, 3% B. Relevant mass spectrometric parameters are provided in Table S2.

### 2.4. Quality Assurance and Quality Control (QA/QC)

To eliminate the influence of background contamination, procedural blanks were included in each batch, totaling 20 samples. No target analytes were detected in any of the blank samples. The limits of detection (LODs) and quantification (LOQs), defined as signal-to-noise ratios (S/N) of 3 and 10, respectively, ranged from 0.0056 to 0.0597 mg/kg and from 0.0188 to 0.199 mg/kg. The recoveries of NEOs in all tea samples ranged from 93.3% to 120%. Calibration curves for all analytes exhibited excellent linearity, with correlation coefficients (R^2^) exceeding 0.999. Detailed information on the LODs, LOQs, and recoveries for the applied analytical method is provided in Table S3.

### 2.5. Health Risk Assessments

Equation (1) was used to calculate the estimated daily intake (EDI, µg/kg Bw/day) to evaluate the chronic risk of NEO ingestion [4]. Equations (2) and (3) were used to calculate each NEO’s chronic hazard quotient (cHQ) and chronic hazard index (cHI), respectively [33,34].

(1) $$EDI=\frac{C\times ADC}{Bw}$$

(2) $$cHQ=\frac{EDI}{RfD}$$

(3) cHI=ΣcHQ

The NEO concentration in tea is represented by C (μg/kg), the average daily tea intake for adults and children is indicated by ADC (g/day), the average body weight is indicated by Bw (kg), and the reference dosage is denoted by RfD (μg/kg/day). For IMI, only the acceptable daily intake (ADI) was available and was therefore used as a substitute for RfD in health risk assessment [1].

Equation (4) was used to determine the estimated short-term intake (ESTI; mg/kg Bw/day) in order to evaluate the acute risk of NEO exposure [33,35]. The acute hazard index (aHI) and acute hazard quotient (aHQ) for each NEO were determined using Equations (5) and (6), respectively [34].

(4) $$ESTI=\frac{HR\times LP}{BW}$$

(5) $$aHQ=\frac{ESTI}{aRfD}$$

(6) aHI=ΣaHQ

The acute reference dosage is represented by aRfD (mg/kg bw), the highest detectable residue level of NEOs in the available tea samples is represented by HR (mg/kg), and large-portion tea intake is indicated by LP (kg).

Elevated acute or chronic exposure risks correlate with higher HQ and HI values. An HQ or HI exceeding 1 signifies a potentially unacceptable risk to human health [29]. Table S4 provides a summary of the parameters used in these calculations.

### 2.6. Data Analysis

GraphPad Prism 10.1.2 was used for data visualization and analysis. Concentrations below the LOD were assigned a value of half the LOD (LOD/2). One-way analysis of variance (ANOVA) and Spearman’s rank correlation were performed using IBM SPSS Statistics 27, with statistical significance set at *p* < 0.05.

## 3. Results and Discussion

### 3.1. Distribution of Neonicotinoid Residue Concentrations in Tea Samples

The detection frequencies (DFs) and concentration ranges (mean, median, minimum, and maximum) of six NEOs observed in the collected tea samples are presented in Table 1. Across all samples, the DFs of NEOs ranged from 8.76% to 57.7%, with DIN being the most frequently detected compound, consistent with previous findings [36]. DIN also exhibited a relatively high mean concentration, ranking second among the six NEOs (DIN = 43.9 ng/g; highest: ACE = 47.4 ng/g). Compared to Li’s 2020 report [29], the DFs of IMI and THI declined from 57.3% to 36.5% and from 30.1% to 12.4%, respectively, yet their widespread presence in tea remains evident. In contrast, DIN’s DF increased significantly from 7.30% to 57.7%, while ACE remained relatively stable (51.0% vs. 49.6%) [29], likely due to its high chemical stability [37]. Notably, THM has been consistently detected in recent years, warranting continued attention. Among the six NEOs, the median concentrations followed the order: DIN (7.31 ng/g) > CLO (0.02 ng/g) > THI (0.01 ng/g) > IMI, ACE, and THM (not detected, N.d.). A study conducted in Taiwan reported a different pattern, with IMI exhibiting the highest DF (92%), followed by DIN (75%), ACE (58%), CLO (42%), and THI (42%). Additionally, IMI showed the highest median residue concentration (35.8 ng/g), followed by DIN (26.9 ng/g) [38]. These regional differences may reflect variations in NEO usage practices and local tea consumption habits.

Of note, two oolong tea samples exhibited particularly high residue levels of NEOs (DIN = 918 ng/g and ACE = 988 ng/g), which may be attributed to specific pesticide usage patterns and processing practices associated with oolong tea production. Table S5 summarizes the maximum residue limits (MRLs) for NEOs established by China, Japan, and the European Union [38]. According to food safety regulations, the detection of residues exceeding the MRL constitutes a regulatory violation. In this study, all analyzed tea samples (*n* = 137) complied with the MRLs set by China and Japan. However, DIN residues in 61 samples (44.5%), IMI residues in 29 samples (21.2%), and ACE residues in 22 samples (16.1%) exceeded the corresponding EU MRLs (10 ng/g, 50 ng/g, and 50 ng/g, respectively), indicating potential health risks.

### 3.2. Contamination Profiles of Six Neonicotinoids

**Figure 1:**
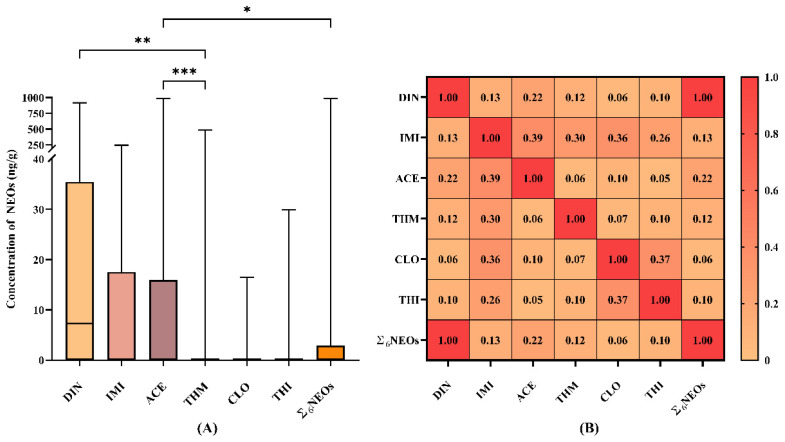
(**A**) Comparison of the measured levels of six NEOs and ∑_6_NEOs across tea samples. (**B**) Spearman’s correlation coefficients for different NEO compounds in all tea samples are shown; boxplot ranges from minimum to maximum values; three horizontal lines represent the 25th, 50th, and 75th percentiles; “*” indicates significance at *p* < 0.05, “**” at *p* < 0.01, and “***” at *p* < 0.001; NEOs: neonicotinoids; DIN: dinotefuran; THM: thiamethoxam; CLO: clothianidin; IMI: imidacloprid; ACE: acetamiprid; THI: thiacloprid; ∑_6_NEOs: sum concentration of all target NEOs.

Figure 1A illustrates the concentration distribution of individual NEOs and the total concentration of target NEOs (∑_6_NEOs) in tea samples. The residue concentration of ACE (47.40 ng/g) was significantly higher than that of THM (4.83 ng/g) (*p* < 0.001) and ∑_6_NEOs (21.28 ng/g) (*p* < 0.05), identifying ACE as the predominant contaminant among the six NEOs detected. This finding highlights the need for further investigation into the health risks and degradation characteristics of ACE. Collectively, ACE, DIN, and IMI accounted for 95.65% of the total NEO residues, indicating that these compounds represent the primary sources of contamination in tea and suggesting their widespread application across different tea plantations. The selection of pesticide formulations likely plays a critical role in determining residue profiles [39]. A 2020 study by Zhang on tea-growing regions in southern China reported ACE, IMI, and THI as the dominant contaminants [40], which differs from the present results. This discrepancy may be attributed to increased DIN usage in recent years and regional variations in pesticide application practices.

Spearman correlation analysis was employed to examine the relationships between various NEOs. (Figure 1B; Table S6). A highly significant positive correlation was identified between DIN and ∑_6_NEOs (r = 1.00, *p* < 0.001), likely attributable to the high DF (57.7%) and median concentration of DIN (7.31 ng/g). Furthermore, IMI exhibited positive correlations with ACE (r = 0.39, *p* < 0.001), THM (r = 0.30, *p* < 0.001), CLO (r = 0.36, *p* < 0.001), and THI (r = 0.26, *p* < 0.001), while a positive correlation was also observed between THI and CLO (r = 0.37, *p* < 0.001). These findings align with those reported by Xiao [28], indicating potential co-application or similar migration behaviors among these compounds, such as using pesticide mixtures and/or multiple pesticide applications during cultivation [36].

### 3.3. Variation in Neonicotinoid Residues Among Different Tea Types

**Figure 2:**
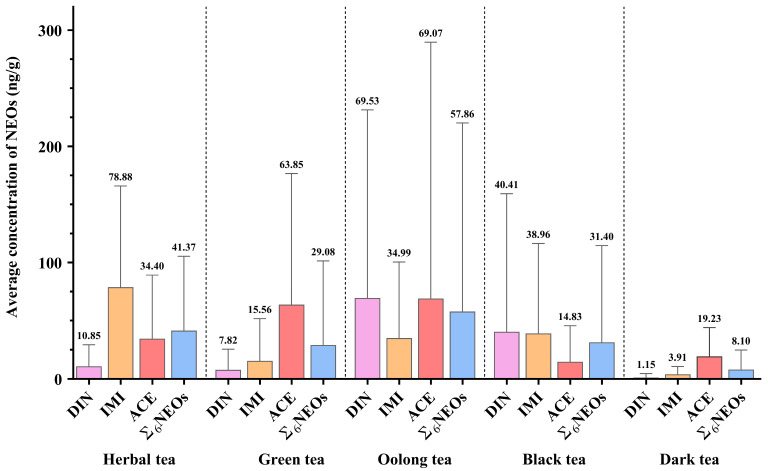
Average concentrations (ng/g) of different NEOs across various tea types; DIN, IMI, and ACE represent dinotefuran, imidacloprid, and acetamiprid, respectively; NEOs refer to neonicotinoids; ∑_6_NEOs represents the sum concentration of all target NEOs.

Figure 2 and Table S7 summarize the NEO residue levels across different types of tea. As noted previously, ACE, DIN, and IMI collectively accounted for 95.65% of total NEO residues, while the remaining NEOs were detected at negligible levels and are therefore not discussed further. Both white tea and green tea are classified as non-fermented teas [41,42]. Green tea undergoes a de-enzyming (or “fixation”) process, which rapidly halts enzymatic activity in the leaves and prevents further oxidation. In contrast, white tea processing is relatively simple, involving only withering and drying. Consequently, white tea lacks distinct representative characteristics compared to other tea types. Green and oolong teas exhibit more distinct and representative processing characteristics. Green tea is processed through fixing, rolling, and drying [42]. And the processes in oolong tea can generally be classified into five steps: withering, fermenting, panning, rolling, and firing [43]. This study investigates the impact of tea processing methods on tea quality, with a focus on tea types that exhibit significant processing differences, such as green tea and oolong tea. Additionally, no residues were detected in any of the white tea samples; thus, the results for white tea were excluded from the primary analysis. Among the five tea types analyzed, the median DIN concentrations followed the order: oolong tea (12.90 ng/g) > black tea (9.32 ng/g) > herbal tea (0.01 ng/g) = green tea (0.01 ng/g) = dark tea (0.01 ng/g). For IMI, the median residue levels were highest in herbal tea (67.10 ng/g), followed by dark tea (1.58 ng/g), oolong tea (0.02 ng/g), green tea (0.02 ng/g), and black tea (0.02 ng/g). ACE exhibited a distinct pattern, with the highest residues in herbal tea (15.00 ng/g), followed closely by green tea (13.20 ng/g), and lower levels in dark tea (10.37 ng/g), oolong tea (0.02 ng/g), and black tea (0.02 ng/g). The overall mean ∑_6_NEOs concentrations ranked as follows: oolong tea (57.86 ng/g) > herbal tea (41.37 ng/g) > black tea (31.40 ng/g) > green tea (29.08 ng/g) > dark tea (8.10 ng/g).

The high average residues of DIN detected in oolong tea align with earlier findings in this study, with oolong tea also exhibiting the highest mean ∑_6_NEOs concentration among all tea types. The octanol–water partition coefficient (log K_ow_) reflects a compound’s lipophilicity, with higher values indicating greater affinity for lipids and potential accumulation in biological membranes or adipose tissues. Similarly, the soil organic carbon–water partition coefficient (log K_oc_) represents the sorption capacity of organic compounds to soil or sediment; higher log K_oc_ values suggest stronger adsorption to soil organic matter, increasing the risk of biological exposure [44,45]. According to Okeke [45], DIN and ACE have log K_ow_ values of –0.55 and 0.80 and log K_oc_ values of 1.41 and 2.30, respectively. Although DIN and ACE are generally hydrophilic, the complex processing steps involved in oolong tea—such as shaking and rolling—may lead to atypical behavior. Physical or chemical changes during these stages could result in enrichment patterns resembling those of lipophilic substances [46]. For example, during the shaking process, mechanical friction damages cell walls, causing tea juice to exude. This may alter the distribution of originally hydrophilic compounds such as DIN and ACE on the leaf surface, and the subsequent drying process could further concentrate these residues [43]. To mitigate pesticide accumulation during oolong tea processing, it is advisable to optimize manufacturing steps by minimizing physical damage and incorporating short-term high-temperature treatments (e.g., infrared deactivation) during fixation or drying stages, thereby selectively degrading pesticides based on their thermal stability.

The highest average IMI residue was detected in herbal tea, likely because many of these products, such as chrysanthemum tea, are derived from floral materials and typically undergo low-temperature drying methods (e.g., hot air drying at 60 °C, natural air-drying, or microwave drying), without exposure to high-temperature fixation or microbially mediated biochemical reactions that could effectively degrade IMI [25]. Additionally, jasmine tea is produced by scenting base tea with fresh jasmine flowers; if the flowers contain IMI residues—either from direct pesticide application or residual contamination—pesticides may transfer to the tea leaves through volatilization–condensation processes during scenting [26]. Furthermore, the mean ∑_6_NEOs concentration in herbal tea (41.37 ng/g) was second only to that in oolong tea, underscoring the necessity for stringent safety regulations for non-traditional tea products.

The relatively low NEO residues detected in black tea may be attributed to its full fermentation (oxidation) process [36]. A study conducted in Yunnan, China, indicated that storage time had no significant effect on pesticide degradation in Pu-erh tea, suggesting that pesticide residues primarily originate during the fresh leaf processing stage [39]. Interestingly, the low DIN and IMI residues observed in dark tea further suggest that refining fresh leaf processing methods, as used for dark tea, may offer strategies for more effectively reducing NEO residues in tea.

Overall, the findings indicate that differences in processing methods among tea types may significantly influence NEO residues, underscoring the need to optimize tea manufacturing processes to mitigate the risk of NEO exposure.

### 3.4. Exposure Risk Assessment

To assess the potential health risks posed by specific NEOs to adults and children, the EDI, ESTI, HQ, and HI were calculated based on the measured concentrations of NEO residues in tea samples [4]. The detailed results are presented in Table 2 and Table 3.

For chronic exposure risk assessment, the median EDI of NEOs in adults ranged from 1.33 × 10^−6^ to 9.75 × 10^−4^, and the median cHQ ranged from 3.81 × 10^−8^ to 3.9 × 10^−5^. These EDI values were substantially lower than their RfD or ADI, and all cHQ values were well below 1, indicating that the chronic exposure risk of NEOs from tea consumption is acceptable for adults. Acute exposure evaluation is also critical for understanding NEO exposure in populations [35]. The ESTI for adults ranged from 2.35 × 10^−11^ to 1.58 × 10^−4^, with corresponding aHQ values ranging from 5.88 × 10^−11^ to 1.58 × 10^−3^, both well within acceptable limits (<1). Although the acute exposure risk from tea appears negligible, further monitoring of NEO residue dynamics during different tea processing stages is recommended. These findings are consistent with those reported by Xiao [28], where median cHQ and aHQ values for adults ranged from 0.00 to 3.85 × 10^−5^ and from 1.25 × 10^−5^ to 1.23 × 10^−3^, respectively.

Risk exposure in children differed from that in adults. In the chronic exposure assessment, the median EDI of NEOs for children ranged from 1.71 × 10^−6^ to 1.25 × 10^−3^, and the median cHQ ranged from 4.88 × 10^−8^ to 4.99 × 10^−5^, approximately 1.28 times higher than those for adults. As a vulnerable population, children warrant greater attention and more tailored scientific guidance regarding NEO exposure from tea consumption. On the other hand, the ESTI for children ranged from 3.51 × 10^−6^ to 2.11 × 10^−4^, with corresponding aHQ values ranging from 5.86 × 10^−6^ to 2.11 × 10^−3^, both remaining well within the acceptable threshold (<1). It is noteworthy that, due to lower body weight and metabolic differences, children’s actual risk may be higher than the calculated estimates [47]. Behavioral factors are also critical and should not be overlooked. A key factor is the actual tea consumption among children, which may vary depending on seasonal changes, time of day, and social contexts. Future assessments should consider developing child-specific physiological correction factors to improve exposure risk evaluation.

Given the frequent use of NEOs as insecticides and their potential cumulative toxicity, the cumulative exposure risks from tea consumption were assessed. To evaluate the cumulative risk of chronic and acute exposure to NEOs through tea consumption, the HI was calculated in this study [28]. Adults and children exhibited median cHI values of 3.99 × 10^−5^ and 5.11 × 10^−5^, respectively, whereas the median aHI values were 1.97 × 10^−3^ and 2.76 × 10^−3^ (Table 2 and Table 3). All values remained within acceptable limits. These findings align with those of Gao [48], who reported a cHI range of 3.81 × 10^−8^ to 3.9 × 10^−5^ in tea samples collected from Hefei, China. Therefore, the health risks connected to NEO residues in tea are considered relatively low for both children and adults based on assessments of chronic, acute, and cumulative exposure. However, it is important to note that the current HI calculations included only six NEOs, excluding potential co-residual pesticides (e.g., pyrethroids) and possible combined exposure effects, which may contribute to additional human health risks. Therefore, it is essential not only to strengthen pesticide management during agricultural application but also to prioritize NEO residue control during tea processing, warranting further investigation.

## 4. Conclusions

In this study, NEOs were widely detected in both tea leaves and their infusions; the DFs for THM, CLO, and THI were relatively small, whereas the concentrations of DIN, IMI, and ACE in some samples exceeded the MRLs established by the European Union. ACE was identified as the predominant NEO residue in tea, together with DIN and IMI; these three compounds are the primary contributors to NEO contamination in tea plantations. IMI exhibited significant correlations with ACE, THM, and other compounds, indicating potential co-application or similar environmental behaviors. Furthermore, tea processing methods significantly influenced NEO residue levels: physical damage during oolong tea processing (e.g., leaf bruising) resulted in the enrichment of DIN and ACE. In contrast, the absence of high-temperature deactivation steps in herbal tea processing led to the highest IMI residues, whereas full oxidation and specific fresh leaf treatments in black and red teas effectively reduced NEO concentrations. Regarding acute, chronic, and cumulative dietary exposure, the health risks associated with NEO residues from tea consumption were generally low for both children and adults. However, greater attention should be given to children’s exposure due to their increased vulnerability.

However, this study focused exclusively on dry tea leaves for exposure characterization and did not include testing of tea infusion liquids. Future research should incorporate infusion testing to provide a more comprehensive assessment of pesticide exposure during tea consumption. Additionally, seasonal variation in pesticide residues was not considered in this study. Future work should include samples collected across different seasons to better understand the dynamic changes in pesticide residue levels.

Overall, this study demonstrates that to enhance food safety initiatives and protect public health, controlling NEO residues in tea requires optimization of processing methods, implementation of precision pesticide application strategies, and consideration of the cumulative effects of multiple pesticide residues.

## Figures and Tables

**Table 1:** Detection frequencies (%) and concentrations (ng/g) of the six selected NEOs in all tea samples.

	DIN	IMI	ACE	THM	CLO	THI	∑_6_NEOs
Total samples (*n* = 137)							
DF(%)	57.7	36.5	49.6	12.4	6.57	8.76	-
Mean (SD)	43.9 (0.02)	30.8 (0.01)	47.4 (0.03)	4.83 (0.01)	0.30 (0.01)	0.43 (0.01)	21.3(0.01)
Min	N.d.	N.d.	N.d.	N.d.	N.d.	N.d.	N.d.
Median	7.31	N.d.	N.d.	N.d.	0.02	0.01	N.d.
Max	918	244	988	482	16.5	29.9	988

“*n*” represents the number of samples; “DF” indicates the detection frequency; and “N.d.” denotes compounds that were not detected. “NEOs” refer to NEO insecticides, including dinotefuran (DIN), imidacloprid (IMI), acetamiprid (ACE), thiamethoxam (THM), clothianidin (CLO), and thiacloprid (THI). “Σ_6_NEOs” represents the sum concentration of the six studied NEOs.

**Table 2:** Results of chronic, acute, and cumulative exposure risk assessments for adults based on the detected NEO concentrations in tea samples.

	Chronic Exposure Risk		Acute Exposure Risk		Cumulative Exposure Risk	
	EDI ^a^ (µg/kg B_W_/day)	cHQ ^a^	ESTI (mg/kg B_W_/day)	aHQ	cHI ^a^	aHI
DIN	9.75 × 10^−4^ (0.00, 1.22 × 10^−1^)	3.90 × 10^−5^ (0.00, 4.88 × 10^−3^)	1.47 × 10^−4^	1.47 × 10^−4^	3.99 × 10^−5^ (0.00, 1.18 × 10^−2^)	1.97 × 10^−3^
IMI	2.67 × 10^−6^ (0.00, 3.25 × 10^−2^)	4.68 × 10^−8^ (0.00, 5.70 × 10^−4^)	2.35 × 10^−11^	5.88 × 10^−11^		
ACE	2.67 × 10^−6^ (0.00, 1.32 × 10^−1^)	3.81 × 10^−8^ (0.00, 1.89 × 10^−3^)	1.58 × 10^−4^	1.58 × 10^−3^		
THM	1.33 × 10^−6^ (0.00, 1.92 × 10^−2^)	2.22 × 10^−7^ (0.00, 3.20 × 10^−3^)	7.71 × 10^−5^	7.71 × 10^−5^		
CLO	2.67 × 10^−6^ (0.00, 2.20 × 10^−3^)	2.72 × 10^−7^ (0.00, 2.24 × 10^−4^)	2.64 × 10^−6^	4.39 × 10^−6^		
THI	1.33 × 10^−6^ (0.00, 3.99 × 10^−3^)	3.33 × 10^−7^ (0.00, 9.97 × 10^−4^)	4.78 × 10^−6^	1.59 × 10^−4^		

EDI, ESTI, cHQ, cHI, aHQ, and aHI represent estimated daily intake, estimated short-term intake, chronic hazard quotient, chronic hazard index, acute hazard quotient, and acute hazard index, respectively; NEOs refer to neonicotinoids; DIN, THM, CLO, IMI, ACE, and THI correspond to dinotefuran, thiamethoxam, clothianidin, imidacloprid, acetamiprid, and thiacloprid, respectively; ^a^ results are expressed as median values (minimum, maximum).

**Table 3:** Results of chronic, acute, and cumulative exposure risk assessments for children based on the detected NEO concentrations in tea samples.

	Chronic Exposure Risk		Acute Exposure Risk		Cumulative Exposure Risk	
	EDI ^a^ (µg/kg B_W_/day)	cHQ ^a^	ESTI (mg/kg B_W_/day)	aHQ	cHI ^a^	aHI
DIN	1.25 × 10^−3^ (0.00, 1.57 × 10^−1^)	4.99 × 10^−5^ (0.00, 6.27 × 10^−3^)	1.96 × 10^−4^	1.96 × 10^−4^	5.11 × 10-5 (0.00, 1.51 × 10^−2^)	2.76 × 10^−3^
IMI	3.41 × 10^−6^ (0.00, 4.16 × 10^−2^)	5.99 × 10^−8^ (0.00, 7.31 × 10^−4^)	5.21 × 10^−5^	1.30 × 10^−4^		
ACE	3.41 × 10^−6^ (0.00, 1.69 × 10^−1^)	4.88 × 10^−8^ (0.00, 2.41 × 10^−3^)	2.11 × 10^−4^	2.11 × 10^−3^		
THM	1.71 × 10^−6^ (0.00, 2.46 × 10^−2^)	2.85 × 10^−7^ (0.00, 4.10 × 10^−3^)	1.03 × 10^−4^	1.03 × 10^−4^		
CLO	3.41 × 10^−6^ (0.00, 2.81 × 10^−3^)	3.48 × 10^−7^ (0.00, 2.87 × 10^−4^)	3.51 × 10^−6^	5.86 × 10^−6^		
THI	1.71 × 10^−6^ (0.00,5.10 × 10^−3^)	4.27 × 10^−7^ (0.00, 1.28 × 10^−3^)	6.38 × 10^−6^	2.13 × 10^−4^		

EDI, ESTI, cHQ, cHI, aHQ, and aHI represent estimated daily intake, estimated short-term intake, chronic hazard quotient, chronic hazard index, acute hazard quotient, and acute hazard index, respectively; NEOs refer to neonicotinoids; DIN, THM, CLO, IMI, ACE, and THI correspond to dinotefuran, thiamethoxam, clothianidin, imidacloprid, acetamiprid, and thiacloprid, respectively; ^a^ results are expressed as median values (minimum, maximum).

## Data Availability

Data are available from the corresponding author by request.

## Supplementary Materials

The following supporting information can be downloaded at: https://www.mdpi.com/article/10.3390/toxics13070550/s1, Table S1: Information on tea samples (*n* = 137); Table S2: Optimized instrument parameters for inductively coupled plasma mass spectrometry; Table S3: Negative multiple reaction monitoring (MRM) transitions for target compounds; Table S4: Parameters used for health risk assessment; Table S5: Standards for the maximum residue level (MRL) of NEOs (ng/g) in China, Japan, and the EU; Table S6: *p*-values of the Spearman’s correlation coefficients for each tea sample’s NEO compounds; Table S7: Concentrations (ng/g) of NEOs in different tea types collected in China [35,49,50,51,52].
