# Supplementary material for: Neonicotinoid Residues in Tea Products from China: Contamination Patterns and Implications for Human Exposure
Source: Toxics. 2025 Jun 29;13(7):550. doi: 10.3390/toxics13070550 (PMC12300606; doi:10.3390/toxics13070550)
Supplement: Supplementary file 1 [file toxics-13-00550-s001.zip › toxics-3644556-supplementary.pdf]

Supporting Information

Neonicotinoid residues in tea products from China: Contamination patterns and implications for human exposure

Yulong Fan [1, 2,†], Hongwei Jin [3, †], Jinru Chen[1], Kai Lin[4], Lihua Zhu[1], Yijia Guo[1], Jiajia Ji[4], Xiaming Chen [1,*]

[1] Public Health Service Center, Bao'an District , Shenzhen, 518105, China

[2] School of Public Health, Sun Yat-Sen University, Guangzhou, 510080, China

[3] Shenzhen Guangming District Center for Disease Control and Prevention, Shenzhen, 518107, China

[4] Shenzhen Center for Disease Control and Prevention, Shenzhen, 518055, China

[†] These authors contributed equally to this work.

[*]Corresponding author:

Chief Physician. Xiaming Chen, E-mail: chenxiam@mail3.sysu.edu.cn

Table S1. Information on tea samples (n=137).

Table S2. Optimized instrument parameters for inductively coupled plasma mass spectrometry.

Table S3. Negative multiple reaction monitoring (MRM) transitions for target compounds.

Table S4. Parameters used for health risk assessment.

Table S5. Standards for the maximum residue level (MRL) of NEOs (ng/g) in China, Japan, and the EU.

Table S6. P-values of the Spearman's correlation coefficients for each tea sample's NEO compounds.

Table S7. Concentrations (ng/g) of NEOs in different tea types collected in China.

Table S1.
Information on tea samples (n=137).

Tea type	Representative tea	Example	Number
Substitutional tea	Non-tea drinks	Jasmine tea, chrysanthemum tea, rose tea	7
Unfermented tea	Green tea	Long jing, Mao Jian, fried green tea	20
Semi-fermented tea	Oolong tea, white tea	Tie Guan Yin, Da Hong Pao, Phoenix Monocotyledon, Gong Mei	74
Fully fermented tea	Black tea	Lapsang Souchong, Dian Hong, Jin Jun Mei, Hong Yu	21
Post-fermented tea	Dark tea	Pu'er, pressed Shou Mei	15

Table S2.

Optimized instrument parameters for inductively coupled plasma mass spectrometry.

Parameter	Value
Desolvation Temperature (℃)	450
Source Temperature (°C)	120
Capillary (kV)	0.75
Cone (V)	30
Source offset (V)	50
Desolvation Gas Flow (L/hr)	900
Cone Gas Flow (L/Hr)	150
Collision Gas Flow (mL/Min)	0.11
Nebuliser（bar）	7

Table S3.

Negative multiple reaction monitoring (MRM) transitions for target compounds.

Compound	Parent ion (m/z)	Product ions (m/z)	Dwell (s)	Cone (V)	CE (V)	DP (V)	Recovery (%)	LOD (ng/g)	LOQ (ng/g)
THM	292.00	181.10	0.003	30	20.62	86.73	101 – 120	1.10E-05	3.66E-05
	292.00	211.10	0.003	30	10.25	86.73			
THM-d_3	294.94	214.00	0.003	30	10.25	88.55			
CLO	250.02	169.00	0.003	30	14.50	80.66	93.3 - 112	4.21E-05	1.40E-04
	250.02	132.00	0.003	30	10.25	80.66			
CLO-d_3	252.94	172.07	0.003	30	10.25	78.24			
IMI	256.10	175.10	0.003	45	16.88	91.58	98.3 - 112	4.21E-05	1.40E-04
	256.10	209.10	0.003	45	14.30	91.58			
IMI-d_4	260.00	213.07	0.003	45	15.01	91.58			
ACE	223.07	126.00	0.003	34	15.31	101.00	94.7 - 110	4.21E-05	1.40E-04
	223.07	56.10	0.003	34	20.11	101.00			
ACE-d_3	225.97	126.05	0.003	34	20.16	101.60			
THI	253.00	126.00	0.003	32	20.37	113.73	103 - 117	1.23E-05	4.10E-05
	253.00	186.00	0.003	32	13.59	113.73			
THI-d_4	256.97	126.05	0.003	32	20.42	110.70			
DIN	203.11	129.00	0.003	15	13.84	68.83	100 – 116	1.23E-05	4.10E-05
	203.11	157.00	0.003	15	10.25	68.83			
DIN-d_3	206.12	132.13	0.003	15	11.11	64.89			

LOD: limit of detection; LOQ: limit of quantification.

Table S4.
Parameters used for health risk assessment.

Compound	*RfD* [a] (μg/kg/day)	*aRfD* [b] (mg/kg bw)	*LP* [c] (g)		*ADC* [d] (g/day)		B_w [e] (kg)	
			adults	children	adults	children	adults	children
DIN	25	1	9.6	3.2	8	2.56	60	15
THM	6	1						
CLO	9.8	0.6						
IMI	57	0.4						
ACE	70	0.1						
THI	4	0.03						

DIN: dinotefuran; THM: thiamethoxam; CLO: clothianidin; IMI: imidacloprid; ACE: acetamiprid; THI: thiacloprid

[a] *RfD*: the reference dose. Except for IMI, for which we used the acceptable daily intake (ADI, μg/kg/day) from the Ministry of Agriculture (MOA) of China (pesticide acceptable daily intake, NY/T2874-2015), the reference dose for all other compounds was the chronic reference dose from the Environmental Protection Agency (EPA) of the United State [49].

[b] *aRfD*: the acute reference dose [35].

[c] *LP*: the large portion. The maximum daily consumption of tea infusion for adults and children was set as 1500 and 500 mL/person/day, respectively, with a corresponding daily consumption of 9.6 and 3.2 g/person/day of dry tea leaves, respectively [50,51].

[d] *ADC*: the average daily consumption of tea. The average daily consumption of tea infusion for adults and children was set as 1250 and 400 mL/person/day, respectively, with a corresponding daily consumption of 8.0 and 2.56 g/person/day of dry tea leaves, respectively [50,51].

[e] *Bw*: the average body weight of adults and 1- to 6-year-old children [52].

Table S5.
Standards for the maximum residue level (MRL) of NEOs (ng/g) in China, Japan, and the EU.

	China	Japan	European Union
DIN	-	25000	10
IMI	500	10000	50
ACE	10000	30000	50
THM	10000	20000	20000
CLO	-	50000	700
THI	-	30000	10000

NEOs: neonicotinoids; DIN: dinotefuran; IMI: imidacloprid; ACE: acetamiprid;THM: thiamethoxam; CLO: clothianidin; THI: thiacloprid.

Table S6.
P-values of the Spearman's correlation coefficients for each tea sample's NEO compounds.

P-values	DIN	IMI	ACE	THM	CLO	THI	$\sum_6$NEOs
DIN	1.000						
IMI	0.120	1.000					
ACE	**0.008**	**> 0.001**	1.000				
THM	0.171	**> 0.001**	0.521	1.000			
CLO	0.514	**> 0.001**	0.260	0.433	1.000		
THI	0.253	**0.003**	0.547	0.244	**> 0.001**	1.000	
$\sum_6$NEOs	**> 0.001**	0.120	**0.008**	0.171	0.514	0.253	1.000

DIN: dinotefuran; IMI: imidacloprid; ACE: acetamiprid;THM: thiamethoxam; CLO: clothianidin; THI: thiacloprid; $\sum_6$ NEOs: sum concentration of all the target NEOs.

Table S7.
Concentrations (ng/g) of NEOs in different tea types collected in China.

	DIN	IMI	ACE	THM	CLO	THI	$\sum_6$NEOs
**Herbal tea (n = 7)**							
Mean	10.85	78.87	37.31	0.01	0.02	0.01	21.18
Min	N.d.	N.d.	N.d.	N.d.	N.d.	N.d.	N.d.
Median	0.01	67.1	15	0.01	0.02	0.01	0.02
Max	38.20	215.00	144.00	0.01	0.02	0.01	215.00
**Green tea (n = 20)**							
Mean	7.82	15.56	63.85	0.01	0.02	0.01	14.54
Min	N.d.	N.d.	N.d.	N.d.	N.d.	N.d.	N.d.
Median	0.01	0.02	13.20	0.01	0.02	0.01	0.02
Max	73.10	130.00	380.00	0.01	0.02	0.01	380.00
**Oolong tea (n = 59)**							
Mean	69.53	34.99	69.07	0.01	0.02	0.01	28.94
Min	N.d.	N.d.	N.d.	N.d.	N.d.	N.d.	N.d.
Median	12.90	0.02	0.02	0.01	0.02	0.01	0.02
Max	918.00	244.00	988.00	0.01	0.02	0.01	988.00
**Black tea (n = 21)**							
Mean	40.41	38.96	14.83	24.63	0.02	0.01	19.81
Min	N.d.	N.d.	N.d.	N.d.	N.d.	N.d.	N.d.
Median	9.32	0.02	0.02	0.01	0.02	0.01	0.02
Max	554.00	228.00	125.00	482.00	0.02	0.01	554.00
**Dark tea (n = 15)**							
Mean	1.15	3.91	19.23	1.74	0.52	2.57	4.86
Min	N.d.	N.d.	N.d.	N.d.	N.d.	N.d.	N.d.
Median	0.01	1.58	10.37	1.06	0.02	0.01	0.02
Max	13.20	26.98	69.68	13.01	6.45	29.90	69.68

N: number; ND: not detected; NEOs: neonicotinoids; DIN: dinotefuran; THM: thiamethoxam; CLO: clothianidin; IMI: imidacloprid; ACE: acetamiprid; THI: thiacloprid; $\sum_6$NEOs: the sum concentration of all target NEOs.

References

49. Zhang, Q.; Lu, Z.; Chang, C.-H.; Yu, C.; Wang, X.; Lu, C. Dietary risk of neonicotinoid insecticides through fruit and vegetable consumption in school-age children. *Environ Int* **2019**, *126*, 672-681, doi:10.1016/j.envint.2019.02.051.
35. Cui, K.; Wu, X.; Wei, D.; Zhang, Y.; Cao, J.; Xu, J.; Dong, F.; Liu, X.; Zheng, Y. Health risks to dietary neonicotinoids are low for Chinese residents based on an analysis of 13 daily-consumed foods. *Environ Int* **2021**, *149*, 106385, doi:10.1016/j.envint.2021.106385.
50. Li, L.; Fu, Q.-L.; Achal, V.; Liu, Y. A comparison of the potential health risk of aluminum and heavy metals in tea leaves and tea infusion of commercially available green tea in Jiangxi, China. *Environ Monit Assess* **2015**, *187*, 228, doi:10.1007/s10661-015-4445-2.
51. Miri, M.; Bhatnagar, A.; Mahdavi, Y.; Basiri, L.; Nakhaei, A.; Khosravi, R.; Eslami, H.; Ghasemi, S.M.; Balarak, D.; Alizadeh, A.; et al. Probabilistic risk assessment of exposure to fluoride in most consumed brands of tea in the Middle East. *Food Chem Toxicol* **2018**, *115*, 267-272, doi:10.1016/j.fct.2018.03.023.
52. El-Nahhal, Y. Pesticide residues in honey and their potential reproductive toxicity. *Sci Total Environ* **2020**, *741*, 139953, doi:10.1016/j.scitotenv.2020.139953.
